# A Scoping Review of Obesity among Indigenous Peoples in Canada

**DOI:** 10.1155/2019/9741090

**Published:** 2019-06-03

**Authors:** Malek Batal, Stéphane Decelles

**Affiliations:** ^1^Nutrition Department, Faculty of Medicine, Université de Montréal, 2405 Ch de la Côte Ste-Catherine, Montréal, Québec, Canada H3T 1A8; ^2^WHO Collaborating Centre on Nutrition Changes and Development (TRANSNUT), Nutrition Department, Université de Montréal, Montreal, Canada H3T 1A8

## Abstract

Indigenous populations in Canada are heavily affected by the burden of obesity, and certain communities, such as First Nations on reserve, are not included in the sampling framework of large national health surveys. A scoping review of ever published original research reporting obesity rates (body mass index ≥ 30), among adult Indigenous peoples in Canada, was conducted to identify studies that help close the Canadian Community Health Survey (CCHS) data gap for obesity prevalence in Indigenous populations in Canada and to make comparisons based on ethnicity, sex, time, and geography. First Nations on reserve with self-reported height and weight had higher rates of obesity (30%–51%) than First Nations off reserve (21%–42%) and non-Indigenous populations (12%–31%) in their respective province or territory, with the exception of Alberta, where rates in First Nations on reserve (30% and 36%) were lower or similar to those reported in First Nations off reserve (38%). First Nations on reserve with predominantly measured height and weight (42%–66%) had higher rates of obesity compared to Inuit in Quebec (28%), Nunavut (33%), and Newfoundland and Labrador (41%), while the rates were similar to those in Inuit in Northwest Territories (49%). Obesity in these large studies conducted among Inuit was based solely on measured height and weight. Studies in First Nations and Inuit alike showed higher prevalence of obesity in women, as well as an increase with time. No recent studies measured the obesity rates for First Nations in Yukon and Northwest Territories and for Métis living in settlements of Northern Alberta. Researchers are encouraged to conduct total diet studies in these regions, and to use existing data to analyze the associations between obesity, road access, latitude, food environment, and traditional food intake, to further inform community planning and development.

## 1. Introduction

Obesity is a significant and potentially modifiable risk factor for non-insulin-dependent diabetes mellitus, cardiovascular disease, many forms of cancer, asthma, gallbladder disease, osteoarthritis, and chronic back pain [1]. In Canada, more than a quarter (28.1% in 2015) of residents aged 18 to 79 years are obese [2], and the rates are the highest in the territories followed by the Atlantic Region and the Prairies. This is particularly true in rural regions in the Atlantic and Prairie provinces [3]. Obesity was also found to be less prevalent among men and women with immigrant status or of a visible minority, women with higher socioeconomic status, and men and women with higher education attainment [4]. However, obesity and associated noncommunicable diseases were found to be much higher among Indigenous peoples in Canada [5].

The Canadian Constitution recognizes three distinct Indigenous groups: First Nations, Métis, and Inuit [6]. According to the 2011 National Household Survey, Indigenous people make up 4.3% of Canada's total population, an increase from 2.8% in 1996. In 2011, 1,400,685 First Nations people were living both on reserve and off reserve throughout the country, with the largest populations in Ontario (ON) (23.6%), British Columbia (BC) (18.2%), and the three Prairie provinces of Alberta (AB) (13.7%), Saskatchewan (SK) (12.1%), and Manitoba (MB) (13.4%). Métis people, with a population of 451,795, represent 5% or more of the populations of SK (5.2%), MB (6.7%), and the Northwest Territories (NT) (8.0%), and the majority of the 59,455 Inuit people were living in a region of Northern Canada named “Inuit Nunangat” stretching from Newfoundland and Labrador (NL) to NT, including Nunavik in Quebec (QC) and the Territory of Nunavut (NU) [7]. For the remainder of the document, the abbreviations (Canadian provinces: BC: British Columbia, AB: Alberta, SK: Saskatchewan, MB: Manitoba, ON: Ontario, QC: Quebec, NB: New Brunswick, NS: Nova Scotia, PE: Prince Edward Island, and NL: Newfoundland and Labrador. Canadian territories: YK: Yukon, NT: Northwest Territories, and NU: Nunavut) for provinces and territories in Canada will be used.

Collectively, Indigenous people are some of the most socioeconomically disadvantaged in Canada. They have a much lower proportion of secondary school graduates compared to the general population (62.0% vs 79.9% in 2011), mean annual income 27% below the Canadian average (29,780.00$ vs 40,650.00$ in 2011) [8, 9], and a significantly higher prevalence of food insecurity (23.1% vs 7.7% in 2012) [10], with even higher rates among First Nations on reserve [11–17] and Inuit in the Inuit Nunangat [18]. These are all factors that may influence individuals' dietary patterns [19, 20] and physical activity [21], and thus, they have a considerable effect on the prevalence of obesity [22]. Geographic remoteness may also be a contributing factor to both food insecurity and obesity among Indigenous peoples due to the high cost of food in Northern communities [23], although this may be mitigated in those who adhere to a traditional lifestyle [24].

Gionet and Roshanafshar used the combined data for 2007–10 cycles of the Canadian Community Health Survey (CCHS) to estimate obesity rates for First Nations off reserve (26%), Métis (22%), Inuit (26%), and non-Indigenous people (16%) in Canada [5]. The CCHS data are intended to be representative of the entire population in Canada, but the sample excludes certain sensitive areas including First Nations reserves, Métis settlements of Northern AB, Inuit living in Nunavik, QC, and James Bay Cree of QC, in addition to other non-Indigenous remote communities and the armed forces. Although the coverage in NU was recently increased to 92% (2013), CCHS 2007–10 surveys were only conducted in the ten largest communities (71% coverage) [25].

Another limitation of the CCHS is that obesity rates are based on self-reported height and weight, which are known to underestimate obesity prevalence compared to measured height and weight [26]. For example, the 2009–11 Canadian Health Measures Survey (CHMS) found an obesity rate of 27% and 25% in men and women, based on measured height and weight [2], while the rates in the CCHS 2010 were 19% for men and 16% for women. The same limitation applies to the obesity rates reported in the Aboriginal Peoples Survey (APS) [27, 28], which is also representative of First Nations off reserve, Inuit, and Métis peoples in Canada.

In addition to the CCHS and APS, many other studies have measured the prevalence of obesity among Indigenous peoples in Canada. A systematic review and meta-analysis published in 2017 aggregated the obesity data from 25 studies conducted between 1990 and 2013 to produce a pooled obesity prevalence of 37%, with a higher prevalence in women than men (41% vs 32%). Analysis by Indigenous group found obesity prevalence to be highest in Métis (42%) and First Nations (41%) and lowest among Inuit (32%) [29]. Although the review included published secondary analyses of national surveys, it did not include reports from the grey literature and did not distinguish between the use of self-reported and measured anthropometric indices.

Moreover, in a 2012 scoping review of obesity studies among the circumpolar Inuit, reviewers examined methods used for measuring obesity in Inuit living in Canada, Greenland, and the United States of America and presented sex-specific prevalence of obesity as measured using body mass index (BMI) and/or waist circumference. They also reviewed the statistical associations between obesity and metabolic risk factors, diet, and socioeconomic status [30]. However, the examination of obesity distribution between and within countries was outside the scope of this review.

The aim of this work is to present an updated and comprehensive review of all available reports of obesity prevalence in adult Indigenous populations in Canada and to describe the findings for each Indigenous group according to ethnicity, sex, time, and geography, with particular emphasis on identifying studies that address limitations in the CCHS sampling framework.

## 2. Materials and Methods

A scoping review of the literature was conducted until February 20, 2019, in accordance with Colquhoun et al.'s 2014 recommendations [31], which are to follow the methodology first put forward by Arksey and O'Malley in 2005 [32] and later updated by Levac et al. in 2010 [33]. In brief, the methodology requires that the reviewers follow 5 stages:

1. Identify the research question clearly, in conjunction with the purpose of the scoping review (*identifying the research question*)
2. Rely on the research question to identify relevant studies and justify any limits to the comprehensiveness of the review (*identifying relevant studies*)
3. Independently select studies and meet regularly to identify any discrepancies, ideally, incorporating a third reviewer to resolve disagreement (*study selection*)
4. Collaboratively determine the nature of the data to extract from the publications, pilot the charting process using five to ten studies, and make modifications as needed throughout the charting process in an iterative manner (*charting the data*)
5. Present results in a numerical order using tables and charts, followed by a thematic analysis related to the study purpose (*collating, summarizing, and reporting the results*)

The Preferred Reporting Items for Systematic reviews and Meta-Analyses extension for Scoping Reviews (PRISMA-ScR) guidelines were followed, which are a set of 20 essential items and 2 optional items that were created to help improve the quality, completeness, and transparency of scoping reviews [34].

### 2.1. Identifying the Research Question

The main research questions for this scoping review are as follows:

1. How do obesity rates in Indigenous populations compare to those in Canada's non-Indigenous population, and how do they compare between Indigenous groups (First Nations on and off reserve, Métis, and Inuit)?
2. How does obesity prevalence compare between Indigenous men and women?
3. Has obesity prevalence changed with time in Indigenous populations?
4. What is the obesity prevalence for each Indigenous group throughout the country?

### 2.2. Identifying Relevant Studies

The following databases, which focus on health and health-related topics, were used to search for studies that have been published on obesity in Indigenous peoples of Canada:

- Biological Abstracts/RRM 1992 to 2013
- CAB Abstracts 1977 to 2018
- EBM Reviews-Cochrane Database of Systematic Reviews 2005 to 2019
- Embase 1974 to February 19, 2019
- Global Health 1973 to 2019 Week 06
- Ovid MEDLINE(R) 1946 to February Week 2 2019
- Web of Science 1945 to 2019

The search terms used in all databases were (aboriginal$ or first nation$ or inui$ or meti$ or indig$) and (weight or body mass index or obes$ or overw$) and Canad$.

Additional sources, especially from the grey literature, were identified through hand searching reference lists of selected papers and through targeted searches of health data for Indigenous peoples (i.e., International Polar Year Inuit Health Survey (IPY-IHS), Nunavik Health Survey Qanuippitaa? How are we? (NHS), Food Mail Program (FMP), First Nations Regional Health Survey (FNRHS), First Nations Food, Nutrition and Environment Study (FNFNES), Nituuchischaayihtitaau: Multi-Community Environment and Health Study in Iiyiyiu Istchee (MCEHSII), and books published by the Centre for Indigenous Peoples' Nutrition and Environment (CINE)).

### 2.3. Study Selection

All references were imported into EndNote X7, duplicates were removed, and the records were screened for the following criteria:

  Inclusion criteria:

- Original research that presents new obesity prevalence data
- Obesity rates are representative of all Indigenous adults in a defined geographic area
- Obesity is reported as BMI ≥ 30
- Full-text articles are written in English or French
- There are no limitations on years of study or publication

  Exclusion criteria:

(i) Given the use of CCHS 2007–10 data as reference points, secondary analysis of data from the CCHS or other national surveys with Indigenous peoples of Canada living off reserve

Though it is recognized that BMI ≥ 30 is not the most ideal indicator for estimating the cardiovascular risk, it was chosen as the only definition of obesity among the inclusion criteria because it is the most widely used method for assessing obesity in human subjects [35], thus allowing for the inclusion of the greatest number of studies. Using this definition of obesity also made qualitative comparisons to the CCHS 2007–10 possible, which was necessary for answering research question #1, stated above.

### 2.4. Charting the Data

The data charting process was conducted independently by the second reviewer, and all ambiguities in regard to the type of data considered for the final selection of publications were discussed with the first reviewer.

In the first step of the review, publications were included or excluded based on their title and abstract. If there was a reason to believe the obesity rates for an adult Indigenous population were reported in the article (e.g., if mentioning obesity, diet, or physical activity in the title or abstract), the article was kept for further review.

During the second round of selection, the “Ratings” function in EndNote X7 was used to categorize studies as follows:

1. Not reporting obesity (1 star)
2. Making secondary use of CCHS or other national surveys in Indigenous peoples off reserve (2 stars)
3. Not being representative of all adults in a defined geographic area (3 stars)
4. Presenting obesity rates using data from a study which has already been retained (4 stars)
5. Being retained for the final article (5 stars)

In cases where many articles meeting all inclusion criteria used the same data to produce obesity estimates (see the category for “4 stars”), the full charting process was performed for the article focusing on obesity the most, and other articles were identified, in EndNote X7, as being in relation to this main article using the “Research Notes” field. In cases where a baseline report was published in the grey literature, this report was given priority over all other publications.

The following information, as agreed upon by both reviewers, was extracted into Excel for the studies retained for the full charting process:

- Name of the study
- Province/territory
- Number of communities in which the study took place
- Geographic area represented
- Factors that might affect representativeness in the determined geographic area
- Age of participants
- Sample size for measured height and weight
- Sample size for reported height and weight
- Number of male participants
- Number of female participants
- Year(s) of data collection
- BMI ≥ 30 (all)
- BMI ≥ 30 (male)
- BMI ≥ 30 (female)

The chart was piloted using six studies that were already known, by reviewers, to report obesity rates in a defined geographic area [11, 36–40]. After piloting the chart, it was modified as needed, based on discussions between the two reviewers. For instance, after the pilot, certain studies were reviewed for which certain inclusion and exclusion criteria affected the representativeness of a sample. For instance, the reviewers agreed that a diabetes screening project could artificially inflate the obesity prevalence in a region, thus rendering results that are not truly representative of the population [41]. The same was true for age eligibility criteria, which may be too restricted to represent the full adult population in the defined area (i.e., women of 20–40 years of age) [42, 43].

After reviewers had made the final selection of articles for the scoping review, these were compared to the studies included in Kolahdooz et al.'s 2017 systematic review and meta-analysis [29] and Galloway et al.'s 2012 scoping review [30], to ensure that no studies were unwillingly excluded from the analysis. This verification confirmed that no eligible studies were left out during the selection process.

### 2.5. Collating, Summarizing, and Reporting the Results

Studies with samples that were representative of all Indigenous individuals in a given province or territory (identified as Studies A, B, C, D, etc.) are presented separately from smaller, community representative studies, henceforth referred to as “Local Studies” (Studies 1, 2, 3, 4, etc.), respecting the order of provinces and territories from west to east. For each Indigenous group, qualitative comparisons were made according to ethnicity, sex, time, and geography. Whenever possible, comparisons were made between studies with a similar methodology.

Studies that were representative of a whole province or territory were also compared to the CCHS 2007–10, which has been used in this scoping review as a reference point for national, provincial, and territorial obesity rates in Indigenous peoples off reserve and non-Indigenous peoples. A data table presenting the CCHS 2007–10 pooled obesity rates in First Nations off reserve, Métis, and Inuit was made publicly available through Statistics Canada's Table 13-10-0457-01 [44]. Although there exist two sources of obesity data for Indigenous peoples living off reserve in Canada, the CCHS 2007–10 was considered as the best source of national data for the purposes of this scoping review because its main objectives are related to health [45], as opposed to the APS, which has the broader goal of describing the social, economic, and health conditions of Indigenous peoples in Canada [46]. Another important reason for choosing the former national data source is the fact that it produces estimates for obesity in non-Indigenous peoples of Canada, allowing wider comparisons.

Most studies conducted in First Nation and Inuit populations used measured height and weight to determine BMI; therefore, it would have been ideal if provincial CCHS 2007–10 data, which are based on self-reported height and weight, were corrected for reporting bias. In 2014, Navaneelan and Janz corrected the CCHS 2003–12 BMI data for the general population of Canada [3] using an equation developed to adjust for the difference between the BMI of CCHS 2005 participants when height and weight were reported and when height and weight were measured. The adjustments accounted for a 35% increase in obesity prevalence, on average [47]. Notwithstanding its relevance in the current study, the equation was not used to adjust the CCHS 2007–10 obesity rates because it required access to the raw data, which was outside the scope of this literature review.

## 3. Results

**Figure 1:**
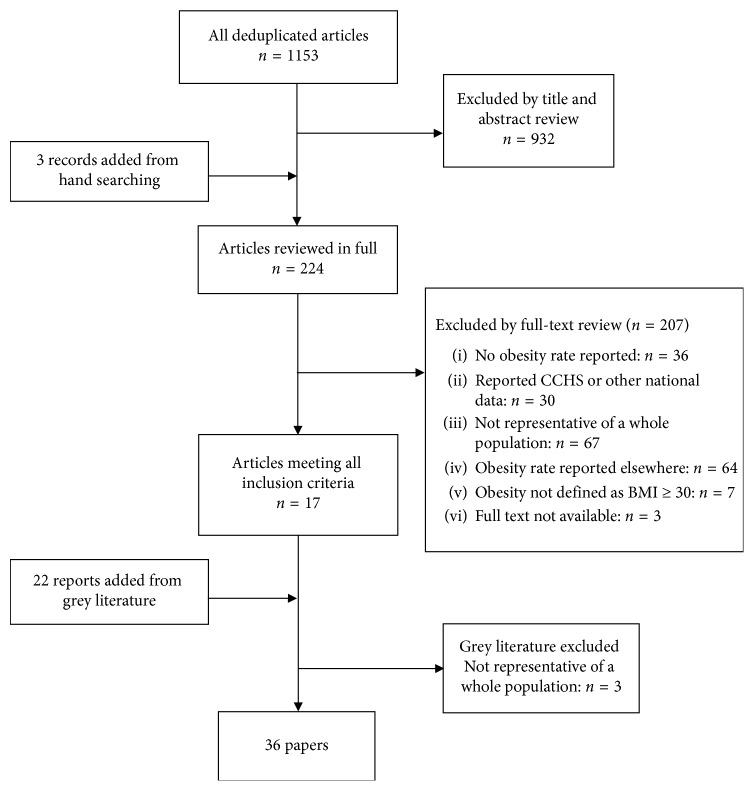
Selection process of papers for review.

The published literature search identified a total of 1984 publications. After removal of duplicates, 1153 unique records remained. Screening by title and abstract led to the exclusion of 929 records. An additional 3 were added following hand searching of reference lists, and 208 were excluded following full-text review, leaving 17 unique studies. Reasons for exclusion included the absence of reported obesity rate (*n*=36), the use of CCHS or other national data (*n*=30) (here are a few noteworthy examples: [28, 48–53]), the sample not being representative of a whole population (*n*=67) as in the case of studies whose participants are restricted to a specific age group [42, 43, 54–56], focus on pregnant women [57–62], diabetes screening initiatives [41, 63–66], and the inclusion of nondiabetic persons only [67, 68]. Studies presenting findings that have already been reported in the 17 unique studies retained for this article (*n*=64) were also excluded, as well as those using a definition of obesity other than BMI ≥ 30 (*n*=7) [69–75] and those for which reviewers were unable to find the full-text article (*n*=3) [76–78]. Of the seven studies using a definition of obesity other than BMI ≥ 30, two presented obesity as BMI ≥ 27 [72, 74], another two combined overweight and obesity (BMI ≥ 25) [73, 75], a further two studies solely presented mean BMI [70, 71], and one last study only used abdominal obesity, which was measured using waist circumference. A further 22 papers were identified in the grey literature search, 19 of which met inclusion criteria. The three papers that were not included in this review presented results from the baseline studies conducted among women of 15–44 years of age in Kugaaruk (NU) [79], Fort Severn (ON) [80], and Kangiqsujuaq (QC) [81] for the Food Mail Program and are thus not representative of the adult population. In sum, 36 unique studies were included in the literature review (Figure 1). The findings are presented according to two of the three Indigenous groups: First Nations and Inuit, because no studies met the inclusion criteria for Métis.

Table 1 presents the national and provincial obesity rates for First Nations, Métis, Inuit, and non-Indigenous people, which were extracted from Statistics Canada's Table 13-10-0099-01 (formerly CANSIM 105-0515) [82]. According to this table, 29% and 27% of First Nations off reserve men and women, 25% and 23% of Métis men and women, 30% and 26% of Inuit men and women, and 18% and 15% of non-Indigenous men and women are obese [44]. Thus, First Nations off reserve, Inuit, and Métis have higher rates of obesity as compared to non-Indigenous people, and little difference exists between men and women for all groups, although males tend to have a slightly higher prevalence than women in each population.

At the provincial/territorial level, the prevalence of obesity was the lowest for Métis and non-Indigenous people in BC (15% and 12%) and QC (20% and 15%). In First Nations off reserve, the lowest rates were found in NT (21%) and QC (21%). Obesity rates for First Nations in NT (21%) and YK (23%) were lower than the average for First Nations off reserve across Canada (28%), while this was not the case for Métis: 31% in NT and 24% in YK vs 24% for Canada. As for Inuit, the lowest obesity rate was in NU (22%).

The regions with the highest obesity rates differed for each group. For non-Indigenous people, the highest rates were in the Atlantic Region (NS: 23%, NB: 24%, and NL: 26%) and the territories NT (22%) and NU (31%), while the rates for First Nations off reserve were highest in the Prairies (SK: 34%, AB: 38%, and MB: 41%) and PE: 42%. The highest rates for Métis were not specific to any particular region (AB: 30%, MB: 30%, NT: 31%, and NB: 32%), while obesity rates for Inuit were highest in NT (36%) and NL (38%).

### 3.1. First Nations

#### 3.1.1. First Nations: Provincial Obesity Rates

*(1) Publications*. Twenty publications reported obesity rates for samples that were representative of all First Nations in a province or territory. Most of the studies reported obesity rates in First Nations on reserve, except Study E, UBC 2007–10, which included First Nations both on and off reserve [83]. The studies cover the YK territory and all 10 provinces (Table 2).

*(2) Measures of Obesity*. Rates of obesity were based on self-reported height and weight in the FNIRHS and FNRHS studies (A–C), which will henceforth be referred to as FN(I)RHS studies, and measured height and weight in Studies E-F. The FNFNES (Study D) used measured height and weight when possible but included reported values when these were not available.

*(3) Differences between Studies within Provinces*. In BC, AB, ON, and QC, more than one study was conducted in the same province during the same period (2008–16). There was a variation in reported obesity rates for studies conducted in BC, AB, and QC, while the difference was slim between those done in ON.

In BC, the rate for Study C, FNRHS 2008–10 (34%), was considerably different from that for Studies D, FNFNES 2008-09 (42%) and E, UBC 2007–10 (49%). This can be explained by the use of self-reported values for height and weight in Study C, use of measured values in Study E, and use of reported values in Study D when measurements were not available. In AB and QC, the explanation for the difference between Studies C and D, FNRHS 2008–10 (36% and 41%) and FNFNES 2013 (42% and 66%), is likely the same.

In contrast, the two most recent ON studies reported comparable obesity rates, even though Study C, FNRHS 2008–10 (48%), used self-reported values for height and weight, while Study D, FNFNES 2011-12 (49%), used reported values only when measurements were unavailable. It is important to keep in mind, however, that values were reported for 2 out of 3 participants in ON for Study D.

*(4) Comparison to CCHS 2007–10*. All recent studies of First Nations on reserve (≥2000) showed obesity rates which are based on self-reported height and weight (Studies A–C, FN(I)RHS) were much higher than the national CCHS 2007–10 results for non-Indigenous people. The differences were between +12% in AB (Study B, FNRHS 2002-03) and +31% in MB (Study B, FNRHS 2002-03) and ON (Study C, FNRHS 2008–10). When compared to First Nations off reserve, the prevalence was higher in most FNRHS studies (+9% to +23%), except in AB, for Studies B and C, FNRHS 2002-03 (−8%) and FNRHS 2008–10 (−2%).

For Studies D and E, FNFNES 2008–16 and UBC 2007–10, in which measured height and weight were predominantly (FNFNES) and strictly (UBC) used to measure obesity, higher prevalence was systematically found in First Nations on reserve compared to non-Indigenous people and First Nations off reserve. The differences with non-Indigenous people ranged from +25% in the Atlantic provinces (Study D, FNFNES 2014) to +51% in QC (Study D, FNFNES 2016). When compared to First Nations off reserve, the differences were between +6% in AB (Study D, FNFNES 2013) and +45% in QC (Study D, FNFNES 2016).

*(5) Sex*. Of the 15 studies providing sex-disaggregated obesity data, 11 reported a higher prevalence of obesity in women. The other 5 studies reported comparable obesity rates for men and women in BC (Study D, FNFNES 2008-09: 44% vs 41%, Study E, UBC 2007–10: 48% vs 49%), ON (Study C, FNFRHS 2008–10: 47% vs 49%), and QC (Study D, FNFNES 2016: 66% vs 65%). This represents 1 out of 6 studies using self-reported height and weight (Studies A–C, FN(I)RHS 1997, 2002-03, and 2008–10), 1 out of 2 studies using measured height and weight only (Study E, UBC 2007–10 and Study F, NCP 1995), and 2 out of 7 studies using self-reported height and weight when measured values are unavailable (Study D, FNFNES 2008–16).

*(6) Time*. According to the FN(I)RHS studies (A–C), for which obesity prevalence is available at two points in time for 5 provinces, obesity rates increased across all these provinces (+2% to +11%), though the increase in BC (+2%) was minimal:

- BC (FNRHS 2002-03 vs 2008–10): increase from 32% to 34%
- AB (FNRHS 2002-03 vs 2008–10): increase from 30% to 36%
- ON (FNRHS 2002-03 vs 2008–10): increase from 39% to 48%
- QC (FNRHS 2002-03 vs 2008–10): increase from 30% to 41%
- NS (FNIRHS 1997 vs FNRHS 2002-03): increase from 38% to 45%.

Obesity rates for First Nations on reserve at the national level increased modestly over time (+4%): 36% were obese in 2002-03 as compared to 40% in 2008–10 [94, 95].

*(7) Geography*. Study D, FNFNES 2008–16, was used for the comparisons based on geography because obesity rates were available for all provinces in Canada. According to the reports, the lowest rates of obesity were in BC (42%) and AB (44%), while the highest rates were in ON (49%), MB (52%), and QC (66%). The results from Studies B and C, FNRHS 2002-03 and FNRHS 2008–10, seem to show a similar pattern between provinces with the exception of QC ranking among the lowest rates in Study B, FNRHS 2002-03.

#### 3.1.2. First Nations: Local Obesity Rates

*(1) Publications*. A total of 11 local studies were representative of one or more communities, without being representative of a whole province or territory. All local studies were conducted with First Nations living on reserve, except Study 4, FNBHS 2002–04, which was conducted with urban First Nations women living in or near Winnipeg. At least one study was found for each of the following provinces: BC, SK, MB, ON, and QC. To our knowledge, no local studies have reported obesity prevalence for First Nations in AB, in the Atlantic Region or the territories (Table 3).

*(2) Measures of Obesity*. In 9 out of 11 publications, the calculation of BMI was based on measured height and weight. The exceptions were Study 3, FNLHP 2012-13, for which height and weight were self-reported for all participants, and Study 7, ZATPD 2005, for which self-reported height and weight were used when measurements were not available, which was the case for 8 individuals.

*(3) Sex*. In all 9 studies where obesity rates for both men and women are available, which made strict use of measured height and weight, obesity rates were higher in women.

*(4) Time*. Three sets of local studies were found to have similar locations but differed in regard to the decade of data collection. All studies used measured height and weight to estimate obesity prevalence, except Study 7, ZATPD 2005, for which self-reported values were used for 8 out of 129 participants. Newer studies reported higher rates for all three sets of comparisons:

- In BC, 27% of Nuxalk Nation of Bella Coola were obese (Study 1, NFNPH 1983) compared to 45% of First Nations in seven Gitxsan villages (Study 2, 2006)
- In First Nations of Northern ON and MB (Study 5, NICDS 1986-87), 36% were obese compared to 48% in Northwestern ON (Study 7, ZATPD 2005)
- In James Bay Cree First Nations, 48% were obese (Study 10, CHS 1991) compared to 73% (Study 11, MCEHSII 2005, 2007–09)

*(5) Geography*. One set of studies in First Nations on reserve was used to determine if differences in obesity rates exist between southern and northern communities within the same province. These studies conducted in QC show that the rate in the southern Algonquin communities of Lac Simon and River Desert (Study 9, 1989 (29%)) was much lower than that in James Bay Cree (Study 10, CHS 1991 (48%)). Both studies only used measured height and weight to estimate obesity prevalence.

### 3.2. Inuit

#### 3.2.1. Inuit: Provincial Obesity Rates

*(1) Publications*. Two sets of publications reported obesity rates for samples that collectively were representative of all Inuit regions (Inuit Nunangat) in Canada (Table 4). Studies A and B, SQHSIN 1992 and NHS 2004, reported obesity prevalence in Nunavik, QC, while Studies C and D, NCP 1998-99 and IPY-IHS 2007-08, reported obesity prevalence in the territories: Nunatsiavut, NL, Inuvialuit Settlement Region, NT, and NU.

*(2) Measures of Obesity*. Rates of obesity were based solely on measured height and weight in all four publications.

*(3) Comparison to CCHS 2007–10*. Results from the most recent studies (≥2000) (B, NHS 2004, and D, IPY-IHS 2007-08) though not perfectly comparable to the CCHS 2007–10 data due to the use of measured rather than self-reported height and weight, show that Inuit in QC, NL, and NT had a higher prevalence of obesity compared to non-Indigenous people (+13%, +15%, and +27%, respectively), while the rates were similar to those in NU (+2%). When comparisons were made with results for Inuit from the CCHS 2007–10, obesity rates were similar (+3%) to those in NL and they were higher for NT (+13%) and NU (+11%). Note that given the lack of obesity rates for QC Inuit in the CCHS, comparisons could not be made for QC.

*(4) Comparison to Study D FNFNES 2008–16*. Study D, FNFNES 2008–16, presented in Table 2, was used for the comparison of obesity rates to Inuit (Study B, NHS 2004, and Study D, IPY-IHS 2007-08) because the prevalence of obesity in these studies was based, predominantly, on measured height and weight and because of the availability of obesity rates for all provinces in Canada. Obesity rates for Inuit in QC (Study B, NHS 2004 (28%)) and NU and NL (Study D, IPY-IHS 2007-08 (33% and 41%)) were lower than those reported in all 7 regions of Study D, FNFNES 2008–16 (BC, AB, SK, MB, ON, QC, and Atlantic Region (42% to 66%)). The obesity rate in NT (49%), however, was about equal to the median obesity rate measured in these reports.

*(5) Sex*. In all 4 studies reporting separate results for women and men, obesity rates were higher in women.

*(6) Time*. In each set of studies, obesity prevalence was measured twice in ten years and was found to increase between study intervals. In QC, obesity prevalence increased from 19% (Study A, SQHSIN 1992) to 28% (Study B, NHS 2004). In the other three regions of the Inuit Nunangat, obesity prevalence increased from 24% (Study C, NCP 1998-99) to 36% (Study D, IPY-IHS 2007-08).

*(7) Geography*. The two most recent studies in Inuit populations (Studies B and D) showed differences in obesity prevalence between regions. The lowest rates were found in QC (Study B, NHS 2004 (28%)), followed by 33% in NU, 41% in NL, and 49% in NT (Study D, IPY-IHS 2007-08).

#### 3.2.2. Inuit: Local Obesity Rates

*(1) Publications*. In total, 5 local studies were found to report obesity rates for Inuit in specific regions that were not a whole province or territory (Table 5). Of these, 4 were conducted in NU and the other in NT. No studies were found for the regions of QC and NL.

*(2) Measures of Obesity*. In 3 out of the 5 publications, the calculation for BMI was based on measured height and weight. In the remaining two publications (Study 1, HFN 2008, and Study 4, HFN 2008), reported height and weight values were used to estimate obesity prevalence when measured values were not available, which was the case for 53 out of 196 participants in Study 1, HFN 2008, and 7 out of 218 individuals in Study 4, HFN 2008.

*(3) Sex*. All 3 studies reporting separate obesity rates for men and women found higher rates in women, including Study 1, HFN 2008, and Study 4, HFN 2008, where obesity prevalence for a quarter of participants or less was based on self-reported height and weight.

*(4) Time*. Study 2 KHAS 1990-91 was conducted 15 or more years earlier than the other local studies. Correspondingly, the obesity prevalence reported in this study was the lowest (29%).

## 4. Discussion

In this scoping review of literature, all original scientific and grey literature with published obesity rates in Canadian First Nations, Métis, and Inuit that are representative of a region or community was reviewed to describe obesity prevalence according to ethnicity, sex, time, and geography. CCHS 2007–10 data were used for comparison with a representative sample of non-Indigenous and Indigenous people off reserve in Canada.

One of the main objectives was to identify studies that address the CCHS 2007–10 sampling gap, which includes First Nations reserves, Métis settlements of northern AB, Inuit living in Nunavik, QC, and James Bay Cree of QC, and 30% of NU. The FNFNES 2008–16 and FNRHS 2008–10 reported obesity prevalence in First Nations on reserve; the NHS (2004) reported obesity rates in Nunavik, QC; the MCEHSII (2005–09) reported obesity rates in James Bay Cree of QC; and the IPY-IHS (2007-08) measured obesity in a representative sample of NU. These studies contribute significantly to key data gaps, yet a few remain.

For First Nations on reserve, recent studies (≥2000) reporting obesity rates based on measured height and weight are lacking in YK and NT. At this point in time, the FNRHS 2008–10, which bases its obesity rates on self-reported height and weight, has conducted surveys in these areas. However, obesity rates are currently not presented in the regional reports. It would be interesting to know if, similarly to First Nations off reserve, lower rates of obesity exist for First Nations on reserve in the territories. Furthermore, it would be important to know how this is possible, despite the higher cost of food and beverages in these areas of Canada [65]. One might hypothesize that it is because of greater reliance on traditional food; however, territory-wide studies measuring traditional food use in YK and NT First Nations are over 20 years old [58]. For the Métis population, no studies have been conducted to measure the obesity rates in Northern AB settlements apart from the Mobile Diabetes Screening Initiative (MDSi) whose results were not included in this scoping review of the literature because participants were referred or self-referred to partake in diabetes screening, which would likely overestimate obesity rates in the population [111]. Total diet studies are urgently needed in these regions to determine obesity rates and identify diet, health, and sociodemographic covariates that may be associated with obesity.

The comparisons made in this scoping review identified important variations in obesity prevalence in relation to ethnicity, sex, time, and geography for First Nations and Inuit. According to the FNFNES reports, First Nations on reserve from BC and AB (42% and 44%) had the lowest rates of obesity, while MB and QC had the highest rates (52% and 66%). As mentioned, similar trends were found in the FNRHS 2002-03 and 2008–10; however, QC rates were among the lowest in Canada, rather than the highest in 2002-03. One reason for this could be that James Bay Cree communities of QC, where some of the highest rates of obesity have been measured in Canada [37], were not part of the communities sampled in the FNRHS 2002-03.

Obesity prevalence was higher in studies with First Nations on reserve using self-reported height and weight compared to that with non-Indigenous people and First Nations off reserve living in the same province. The only exceptions were for AB in the FNRHS studies conducted in 2002-03 and 2008–10, which seem to show lower or similar rates among First Nations on reserve compared to First Nations off reserve. In support of this observation, the difference in obesity rates in AB between the FNFNES study (44%) and the CCHS 2007–10 First Nations off reserve (38%) was relatively small (+6%) despite the fact that most participants (512 out of 560) in Study D, FNFNES 2014, provided measured height and weight data. One factor that could contribute to the lower or equal obesity rates in First Nations on and off reserve is the accessibility to roads leading to main service centres for First Nations communities. According to the First Nations Profile made available through the Indigenous and Northern Affairs Canada (INAC) website, only 3 out of the 47 communities in AB (geographic data were not available for 1 community in AB) (6%) do not have year-round road access to a service centre. In contrast, 6 of the 39 communities in QC (geographic data were not available for 2 communities in QC) (15%) do not have year-round access to a service centre in addition to the 6 other communities (15%) that are situated over 350 km from the nearest service centre [111]. AB First Nations on reserve may thus have access to a variety of reasonably priced foods similarly to First Nations off reserve in this same province. Although few community food assessments have been conducted in Indigenous communities, the results of one study showed that prices in an isolated Northwestern Ontario First Nation fluctuate greatly depending on whether ice roads were used to ship food in the winter, or whether food needed to be flown in. Community members often needed to visit other communities to stock up on groceries because food in their community, especially perishables, was expensive and of low quality. In parallel, convenience stores offer a variety of nutrient-poor, energy-dense foods during and after grocery store hours of operation [112].

Use of traditional foods through hunting, fishing, gathering, or sharing is common for resiliency against food insecurity in Indigenous communities. On days when traditional foods are eaten, nutrient intakes tend to be more favourable to health in First Nations [11, 12, 15–17] and Inuit [113]. Furthermore, regular use of the Inuit language, which is an indicator of the preservation of tradition, was inversely related to obesity prevalence [24]. Regrettably, traditional food use has significantly diminished as Indigenous communities are undergoing a nutrition transition toward a greater reliance on market foods, which consist mainly of ultraprocessed foods [36, 114]. Climate change, economic barriers, the presence of an active hunter in the household, and changing food preferences are commonly mentioned as having an impact on traditional food intake of Inuit [18] and First Nations communities [11–17]. It was found that the proportion of calories coming from ultraprocessed food for First Nations on reserve in provinces west of QC (54%) exceeded those found in the general Canadian population (48%). First Nations who were from BC (50%), who ate traditional food (40%), and who received pension or senior benefits (47%) were least likely to consume ultraprocessed food items [115].

As stated in the FNFNES report for BC First Nations on reserve, 79 g of traditional food per person was consumed each day [11]. No other region in Canada came close to this level of traditional food consumption, with ON in distant second (43 g/person/day) [17] and the Atlantic Region consuming the least (24 g/person/day) [15]. Meanwhile, Inuit in NU consume triple the quantity of traditional food eaten in BC First Nations with fresh caribou meat alone (208 g/person/day), not to mention their important intakes of other traditional foods such as Arctic char (113 g/person/day) and dried caribou meat (125 g/person/day).

On average, the calories coming from traditional food for Inuit men and women under 40 years of age were 15% and 11% in NU [116], 13% in QC [117], 8% and 6% in NT [118], and 3% in NL [119]. In QC and NU, where traditional food intake was the highest, Inuit had the lowest obesity rates: 28% and 33%, compared to 41% in NL and 49% in NT. In the latter two regions, obesity rates were also considerably higher than those in non-Indigenous populations (+15% in NL and +27% in NT), compared to NU (+2%), though caution must be used when interpreting these differences due to use of measured height and weight as opposed to self-reported height and weight in the CCHS 2007–10. Nonetheless, it is interesting to note that the magnitude of difference in obesity rates between Inuit and non-Indigenous people of NU is considerably smaller than that observed in NL and NT. When compared to First Nations on reserve, Inuit in 3 out of 4 regions (QC, NU, and NT) had lower prevalence of obesity than that in all 7 regions of the FNFNES 2008–16, with prevalence considerably lower in QC and NU Inuit. As for differences observed with Inuit in the CCHS 2007–10, the similar rate of obesity (+3%) in NL may be the result of varying sampling techniques between the two studies, notably the opportunity for IPY-IHS 2007-08 to visit more northern and isolated regions in order to be representative of the Inuit Nunangat. As demonstrated on the maps created by Inuit Tapiriit Kanatami (ITK), the Inuit Nunangat represents the most northern parts of NT and NL [120]. However, it is difficult to explain the differences in obesity prevalence between the CCHS 2007–10 and IPY-IHS 2007-08 Inuit of NT (+13%) and NU (+11%), given the difference in methods used to estimate obesity prevalence (measured vs self-reported) and the lack of sample description in the CCHS 2007–10. According to the user guide, the CCHS 2007–10 was limited to the 10 largest communities in NU; however, no information was given to explain whether Inuit in NT and NL resided in the Inuvialuit Settlement Region and Nunatsiavut or not [25].

In regard to time, studies conducted with Inuit clearly show a trend toward increasing obesity rates. Obesity increased from 19% to 28% in QC and 24% to 36% in NL, NU, and NT over a 10-year period. Interestingly, Galloway et al. [121] conjured up the obesity rates (BMI ≥ 30) for a study conducted between 1982 and 1984 in Inuit of QC, which did not meet inclusion criteria for this scoping review because the original authors reported mean BMI rather than obesity prevalence [71]. Obesity rates for men and women reported in that literature review were 14% and 23%, respectively [105]. If these rates are in fact true, they are very similar to those that were reported about 10 years later, in the 1992 Santé Quebec Health Survey among the Inuit of Nunavik (15% for men and 24% for women) [105]. The considerable increase in obesity prevalence between 1992 and 2004 among Inuit of QC in comparison to the previous decade could mean that the turning point in the acceleration of the obesity epidemic in this population was likely around the end of the 1990s. This is about a decade later than that in the general Canadian population, as demonstrated in a recent literature review presenting obesity rates for the whole country from 1978 to 2009 [122]. According to that review, obesity in Canada increased from 6.2% in 1985 to 13.1% in 1994 (+6.9%), which represents the greatest 10-year spike in obesity since 1978. This happens to coincide with the signing of the Canada-United States Free Trade Agreement (CUSFTA) in 1989 and the North American Free Trade Agreement (NAFTA) in 1994 which contributed significantly to the increased availability and accessibility to high-fructose corn syrup in Canada, among other ultraprocessed food, which has been associated with a significant increase in calories consumed in the Canadian population [123]. The fact that these types of foods did not become available as quickly in relatively inaccessible places in Canada such as Nunavik might explain why the obesity rates observed in this region (+1%) between 1982–84 and 1992 remained stable. On the contrary, the difference documented in Nunavik (+9%) between 1992 (19%) and 2004 (28%) is quite large in comparison to the increase in obesity observed in Canada (+2.4%) between 1994 (13.1%) and 2004 (15.5%). This could be an indicator of Nunavik residents' increased access, during this decade, to the kind of foods that were made available through CUSFTA and NAFTA. Commensurably, the magnitude of difference in obesity prevalence among Inuit in NL, NU, and NT (+12%), which are regions that are as geographically remote, if not more, than Nunavik, QC, is also much greater between 1998-99 (24%) and 2007-08 (36%) than in Canada (+2.4%) between 1998 (14.5%) and 2007 (16.9%).

According to the FNRHS 2002-03 and 2008–10 cycles, a 4% increase was observed nationally in First Nations on reserve during this 6- to 8-year period, with higher increases observed in certain provinces such as ON (+9%) and QC (+11%). Local studies conducted in First Nations of Northwestern ON and James Bay Cree First Nation of QC support these findings. However, our understanding of the changes in obesity prevalence with time in First Nations on reserve is limited due to the lack of access to obesity rates for each province surveyed in the FNRHS 2002-03 and 2008–10 cycles, as well as a lack of reporting on obesity prevalence in the most recent FNRHS 2015-16 cycle [124]. To our knowledge, no national data exist on the prevalence of obesity in First Nations on reserve prior to 2002, with the exception of the FNRHS pilot study in 1997 (which was then called FNIRHS) in which obesity was only measured in 3 of the 13 Canadian provinces and territories: NS, MB, and Labrador [125]. Nonetheless, the 4 percentage point increase in obesity remains slightly higher in comparison to the difference observed for Canada as a whole (+2.5%), between 2003 (15.2%) and 2009 (17.9%) [122].

Finally, though CCHS 2007–10 data showed more or less equal prevalence of obesity in men and women with a tendency for higher obesity prevalence in men, almost all studies conducted with Indigenous populations showed that women were more likely than men to be obese, regardless of whether measured or self-reported data were used to estimate obesity prevalence. The higher rate of food insecurity in Indigenous communities and greater effect of food insecurity on women's weight could explain these sex-based differences [126]. In Canada, lower socioeconomic status (SES) is associated with higher BMI in women, but not in men [4], which is particularly alarming given the bulk of evidence showing that a mother's weight during pregnancy is a significant determinant of her offspring's weight at birth and later in life (fetal metabolic programming) [127]. A greater understanding of the role of Indigenous women's experiences of food insecurity on health outcomes for themselves and their children is urgently needed.

## 5. Limitations

The scoping review was conducted adhering, as closely as possible, to the recommendations made by Colquhoun et al. in 2014 [31]. However, due to lack of time and financial resources, the charting process was mainly conducted by the second reviewer. Therefore, the recommendation for two reviewers to conduct the charting process independently was not followed. Nevertheless, both reviewers met regularly to discuss any and all ambiguities regarding the best possible criteria to determine if a study is retained or not, notably in the identification of factors that can affect the representativeness of a study. Moreover, the inclusion of a third reviewer, which is strongly suggested by Colquhoun et al. in 2014, was not deemed necessary because there was no disagreement between reviewers, on the criteria used for final inclusion of studies.

Other limits to this review included the use of BMI as the only marker of obesity, given that people with BMI < 30 can be at risk of coronary heart disease as well if they have an accumulation of ectopic fat in and around the organs. Waist-to-height ratio [35] and combined BMI-waist circumference cutoffs based on the risk of disease [128] have been recommended as methods to improve the identification of obesity-related health risks but have not been applied in any studies in Indigenous populations. Use of waist circumference alone, which is more accurate than BMI for predicting the cardiovascular risk [35], has been used to a lesser extent than BMI in studies conducted in Indigenous populations of Canada and was, more often than not, presented in addition to BMI. Hence, obesity measured as BMI ≥ 30 was regarded as the most inclusive main outcome measure for this literature review, even though relying solely on this indicator has led to the exclusion of 7 publications.

Another limit concerning the use of BMI is whether or not it is a useful measure of obesity, particularly for Inuit. Firstly, Inuit tend to have a lower cardiovascular risk than people of European descent, at a given BMI [121]. Furthermore, some researchers have suggested that observed BMI may overestimate the prevalence of obesity in Inuit as a result of their proportionally shorter leg length [129], which has led to the use of sitting height-to-standing height ratio to adjust their BMI [130]. That being said, recent analyses using the IPY-IHS 2007-08 data have placed doubt upon the above-stated conclusions [131, 132], and authors have suggested the use of hypertriglyceridemic-waist phenotype to more accurately estimate the cardiovascular risk in Inuit populations [132]. Given the ongoing discussion concerning the proper way of predicting the cardiovascular risk in Inuit and the almost ubiquitous use of noncorrected BMI and its thresholds in studies conducted in Indigenous and non-Indigenous populations of Canada, BMI was retained as the main comparison variable in this scoping review.

Lastly, the limits of using CCHS 2007–10 results for obesity as reference points were the reliance on self-reported height and weight, which are known to underestimate obesity prevalence compared to measured height and weight, especially in women [3]. Though caution was used when reporting differences in obesity rates between the CCHS 2007–10 and provincial studies in First Nations and Inuit using measured data, the preferable method would have been to use the CCHS 2007–10 raw data to adjust each participant's BMI as was done for CCHS data in the report by Navaneelan and Janz in 2014 [3]. Nevertheless, data manipulation was outside the scope of this literature review.

## 6. Conclusion

It is hoped that this review will help researchers and policy-makers make sense of the studies reporting on obesity in Indigenous populations of Canada and enable them to make informed decisions in regard to obesity-related surveillance and intervention. More specifically, data gaps were recognized for First Nations in NT and YK and Métis settlements in Northern AB. Also, obesity rates were found to increase with time among First Nations and Inuit, and populations with highest obesity rates were identified as women, First Nations on reserve in MB and QC, and Inuit in NL and NT. With these results in mind, we underline the importance of filling the knowledge gaps for First Nations on reserve living in the territories as well as Métis living in the northern AB settlements and conducting regular monitoring of total diet and health studies in First Nations, Métis, and Inuit. We also encourage the leaders of national studies to analyze the relation between obesity, road access, latitude, the food environment, and traditional food intake, to further inform community planning and development.

## Figures and Tables

**Table 1:** Obesity estimates in Canadian adult (>18 years) Indigenous and non-Indigenous populations based on aggregate, age-standardized, Canadian Community Health Survey (CCHS) data from 2007 to 2010 (Statistics Canada's Table 13-10-0099-01) [44].

Province	Sex	% obesity_(reported)_			
		First Nations (off reserve)	Métis	Inuit	Non-Indigenous people
Canada	Both sexes	28	24	28	16
	Male	29	25	30	18
	Female	27	23	26	15

British Columbia (BC)	Both sexes	22	15	F	12
	Male	25	17^E^	F	14
	Female	19^E^	12^E^	F	10

Alberta (AB)	Both sexes	38	30	F	18
	Male	41	29	F	19
	Female	37	30	F	16

Saskatchewan (SK)	Both sexes	34	24	F	21
	Male	36	26	F	23
	Female	33	22	F	19

Manitoba (MB)	Both sexes	41	30	F	20
	Male	33^E^	32	F	20
	Female	42	29	F	19

Ontario (ON)	Both sexes	29	23	F	17
	Male	33	24	F	18
	Female	25	23^E^	F	15

Quebec (QC)	Both sexes	21^E^	20	F	15
	Male	24^E^	20^E^	F	16
	Female	19^E^	22^E^	F	14

New Brunswick (NB)	Both sexes	28^E^	32^E^	F	24
	Male	F	F	F	25
	Female	43^E^	F	F	24

Nova Scotia (NS)	Both sexes	22^E^	23^E^	F	23
	Male	F	25^E^	F	23
	Female	27^E^	21^E^	F	23

Prince Edward Island (PE)	Both sexes	42^E^	F	F	21
	Male	F	F	F	22
	Female	F	F	F	21

Newfoundland and Labrador (NL)	Both sexes	28	21^E^	38	26
	Male	26^E^	28^E^	44^E^	28
	Female	30^E^	F	F	23

Yukon (YK)	Both sexes	23	24^E^	F	19
	Male	20^E^	F	F	20
	Female	25^E^	F	F	18

Northwest Territories (NT)	Both sexes	21	31	36^E^	22
	Male	16^E^	42^E^	F	24
	Female	26	20^E^	44	20

Nunavut (NU)	Both sexes	F	F	22	31
	Male	F	F	24	26
	Female	F	F	20	39^E^

^E^Health Canada urges to “use with caution.” F: Health Canada states that data are “too unreliable to publish.”

**Table 2:** Obesity prevalence in provincial studies conducted among First Nations.

#	Provincial studies (year)	Province	Population	Age	Measured height and weight	Self-reported height and weight	*n* (M)	*n* (F)	Year of data collection	Definition of obesity	Obesity prevalence (%)			Reference	Difference compared to CCHS 2007–10	
											All	M	F		Non-Indigenous people	First Nations off reserve
A	FNIRHS 1997	Nova Scotia	FN on reserve in 9 communities	18+	0	±523	—	—	1997	BMI ≥ 30	38	33	42	[40]	N/A	N/A

B	FNRHS 2002-03	British Columbia	FN on reserve in 39 communities	18+	0	±712	—	—	2002-03	BMI ≥ 30	32	—	—	[84]	+20	+10
		Alberta	FN on reserve in 9 communities	18+	0	591	—	—	2002-03	BMI ≥ 30	30	—	—	[85]	+12	−8
		Manitoba	FN on reserve in 26 communities	18+	0	±3301	1485	1815	2002-03	BMI ≥ 30	51	42	58	[86]	+31	+10
		Ontario	FN on reserve in 29 communities	18+	0	552	358	322	2002-03	BMI ≥ 30	39	—	—	[87]	+22	+10
		Quebec	FN on reserve in 23 communities	18+	0	±1949	—	—	2002-03	BMI ≥ 30	30	28	33	[88]	+15	+9
		Nova Scotia	FN on reserve in 13 communities	18+	0	±482	235	247	2002-03	BMI ≥ 30	45	40	50	[89]	+22	+23

C	FNRHS 2008–10	British Columbia	FN on reserve in 36 communities	18+	0	±1393	632	761	2008–10	BMI ≥ 30	34	29	40	[90]	+22	+12
		Alberta	FN on reserve in 16 communities	18+	0	±714	361	353	2008–10	BMI ≥ 30	36	—	—	[91]	+18	−2
		Ontario	FN on reserve in 24 communities	18+	0	±1500	654	846	2008–10	BMI ≥ 30	48	47	49	[92]	+31	+19
		Quebec	FN on reserve in 21 communities	18+	0	±1364	—	—	2008–10	BMI ≥ 30	41	—	—	[93]	+26	+20

D	FNFNES	British Columbia	FN on reserve in 21 communities	19+	255	637	372	525	2008-09	BMI ≥ 30	42	44	41	[11]	+30^†^	+20^†^
		Alberta	FN on reserve in 10 communities	19+	512	48	220	340	2013	BMI ≥ 30	44	37	49	[16]	+26^†^	+6^†^
		Saskatchewan	FN on reserve in 14 communities	19+	882	87	313	656	2015	BMI ≥ 30	48	40	51	[14]	+27^†^	+14^†^
		Manitoba	FN on reserve in 9 communities	19+	270	342	215	397	2010	BMI ≥ 30	52	42	58	[12]	+32^†^	+11^†^
		Ontario	FN on reserve in 18 communities	19+	414	869	504	775	2011-12	BMI ≥ 30	50	45	54	[15]	+33^†^	+21^†^
		Quebec	FN on reserve in 10 communities	19+	453	62	145	370	2016	BMI ≥ 30	66	66	65	[13]	+51^†^	+45^†^
		Atlantic Region	FN on reserve in 11 communities in NB, NS, and NL	19+	824	121	344	601	2014	BMI ≥ 30	48	44	50	[15]	+25^†‡^	+18^†‡^

E	UBC	British Columbia	FN on and off reserve in 22 locations	18–77	759	0	182	577	2007–10	BMI ≥ 30	49	48	49	[83]	+37^†^	+27^†^

F	NCP	Yukon	FN on reserve in 10 communities	20+	375	0	177	198	1995	BMI ≥ 30	14	10	17	[36]	N/A	N/A

FNIRHS: First Nations and Inuit Regional Health Survey; FNRHS: First Nations Regional Health Survey; FNFNES: First Nations Food, Nutrition and Environment Study; UBC: University of British Columbia; NCP: Northern Contaminants Program. ±Sample size for respondents who accepted to give height and weight data was not available. Sample size was only available for all study respondents. ^†^Reviewers urge caution in the interpretation of differences between studies where the provincial rates of obesity were based, at least in part, on measured height and weight, because the CCHS 2007–10 results to which they are compared were based on self-reported height and weight. ^‡^Rates from Atlantic Canada were compared to the CCHS 2007–10 average for NB, NS, PE, and NL.

**Table 3:** Obesity prevalence in local^£^ studies conducted among First Nations.

#	Local studies (year)	Province	Population	Age	Measured height and weight	Self-reported height and weight	*n* (M)	*n* (F)	Year of data collection	Definition of obesity	Obesity prevalence (%)			Reference
											All	M	F	
1	NFNPH	British Columbia	Nuxalk Nation of Bella Coola	20+	166	0	84	82	1983	BMI ≥ 30	27	23	31	[96]
2	—	British Columbia	FN in 7 Gitxsan villages in Northwestern BC	18+	393	0	185	208	2006	BMI ≥ 30	45	—	—	[97]
3	FNLHP	Saskatchewan	FN in 2 communities	17–85	0	874	428	446	2012-13	BMI ≥ 30	38	—	—	[98]
4	FNBHS	Manitoba	Urban FN women near Winnipeg	25–75	206	0	0	206	2002–04	BMI ≥ 30	—	—	48	[99]
5	NICDS and SLHDP	Manitoba and Ontario	Oji Cree from 7 communities in Northern Ontario and Manitoba	18+	1180	0	519	661	1986-87	BMI ≥ 30	36	—	—	[100]
6	SLHDP	Ontario	Sandy Lake FN	20+	548	0	210	275	1993–95	BMI ≥ 30	36	30	41	[39]
7	ZATPD	Ontario	Ojibway/Oji Cree from 9 communities in Northern-Western Ontario	18+	121	8	43	78	2005	BMI ≥ 30	48	—	—	[101]
8	—	Ontario	Wapekeka and Kasabonika FN	19+	72	0	31	41	2007	BMI ≥ 30	65	61	68	[102]
9	—	Quebec	Algonquin from River Desert and Lac Simon	15+	621	0	267	354	1989	BMI ≥ 30	29	21	34	[103]
10	CHS	Quebec	Cree (Eeyou Istchee) in 9 Eastern James Bay communities	18–74	943	0	—	—	1991	BMI ≥ 30	48	38	57	[104]
11	MCEHSII	Quebec	Cree (Eeyou Istchee) in 7 Eastern James Bay communities	19+	803	0	210	365	2005, 2007–09	BMI ≥ 30	73	64	77	[37]

^£^Local Studies had samples that were representative of all First Nations in one or more communities without being representative of a whole province or territory. NFNPH: Nuxalk Food and Nutrition Program for Health; FNLHP: First Nations Lung Health Project; FNBHS: First Nations Bone Health Study; NICDS: Northern Indians Chronic Disease Study; SLHDP: Sandy Lake Health and Diabetes Project; ZATPD: Zhiiwaapenewin Akino'maagewin: Teaching to Prevent Diabetes; ^7^MCEHSII: Nituuchischaayihtitaau: Multi-Community Environment and Health Study in Iiyiyiu Istchee.

**Table 4:** Obesity prevalence in provincial studies conducted among Inuit.

#	Provincial studies (year)	Province	Population	Age	Measured height and weight	Self-reported height and weight	*n* (M)	*n* (F)	Year of data collection	Definition of obesity	Obesity prevalence (%)			Reference	Difference compared to CCHS 2007–10	
											All	M	F		Non-Indigenous	Inuit
A	SQHSIN	Quebec	Inuit in all 14 Nunavik villages	18–74	270	0	—	—	1992	BMI ≥ 30	19	15	24	[105]	N/A	N/A

B	NHS	Quebec	Inuit in all 14 Nunavik villages	18–74	925	0	—	—	2004	BMI ≥ 30	28	25	31	[106]	+13^†^	N/A

C	NCP	Nunavut and Northwest Territories	Inuit in 18 communities throughout the Inuit Nunangat (not including Nunavik)	20+	375	0	289	272	1998-99	BMI ≥ 30	24	18	30	[36]	N/A	N/A

D	IPY-IHS	Nunavut, Northwest Territories, and Labrador	Inuit in 36 communities throughout the Inuit Nunangat (not including Nunavik)	18+	2178	0	837	1341	2007-08	BMI ≥ 30	36	26	41	[24]	N/A	N/A
		Labrador	Inuit in 5 communities in Nunatsiavut	18+	260	0	—	—	2007-08	BMI ≥ 30	41	—	—	[24]	+15^†^	+3^†^
		Northwest Territories	Inuit in 6 communities in the Inuvialuit Settlement Region	18+	264	0	—	—	2007-08	BMI ≥ 30	49	—	—	[24]	+27^†^	+13^†^
		Nunavut	Inuit in 25 communities in Nunavut	18+	1654	0	—	—	2007-08	BMI ≥ 30	33	—	—	[24]	+2^†^	+11^†^

SQHSIN: Santé Quebec Health Survey among the Inuit of Nunavik; NHS: Nunavik Health Survey Qanuippitaa? How are we? NCP: Northern Contaminants Program; IPY-IHS: International Polar Year Inuit Health Survey. ^†^Reviewers urge caution in the interpretation of differences between studies where the provincial rates of obesity were based, at least in part, on measured height and weight, because the CCHS 2007–10 results to which they are compared were based on self-reported height and weight.

**Table 5:** Obesity prevalence in local^£^ studies conducted in Inuit populations.

#	Local studies (year)	Province	Population	Age	Measured height and weight	Self-reported height and weight	*n* (M)	*n* (F)	Year of data collection	Definition of obesity	Obesity prevalence (%)			Reference
											All	M	F	
1	HFN	Northwest Territories	Inuit in 3 remote communities of the Inuvialuit Settlement Region including Inuvik	19–84	143	53	48	148	2008	BMI ≥ 30	44	40	46	[107]
2	KHAS	Nunavut	Inuit in 8 communities in the Keewatin region	18+	238	0	105	133	1990-91	BMI ≥ 30	29	—	—	[100]
3	—	Nunavut	Inuit in a Baffin community	18+	48	0	11	37	2005	BMI ≥ 30	58	27	68	[108]
4	HFN	Nunavut	Inuit in 3 communities including Cambridge Bay	19–89	211	7	38	180	2008	BMI ≥ 30	44	34	46	[109]
5	—	Nunavut	Inuit of Repulse Bay	18+	165	0	74	91	≥2008	BMI ≥ 30	37	—	—	[110]

^£^Local Studies had samples that were representative of all Indigenous people in one or more communities without being representative of a whole province or territory. HFN: Healthy Foods North; KHAS: Keewatin Health Assessment Study. ≥2008: Year of data collection is not specified in this publication; however, the authors refer the Inuit Health Study as a previous study, thus implying that data collection took place after 2008.
